# Female Medical Education

**Published:** 1856-11

**Authors:** 


### *Female Medical Education.*

We have received, among a number of other announcements, the circular of the "Female Medical College of Pennsylvania," located in Philadelphia. It seems to us that a word of comment on the arguments advanced by the friends of similar institutions may not be amiss at the present time. The corporators of this Philadelphia institution make, in their circular, an appeal for an endowment; and put forth briefly their claims to the liberality of the public at large. It is their fashion to regard their project as a "*cause*" that is an engine of modern progress. They commence their appeal thus: "The corporators of the Female Medical College of Pennsylvania appeal to the friends of humanity in behalf of this institution and the cause it represents. They regard the medical education of women as a necessity of the age, and a way-mark of the advancement of a refined civilization."

It would seem from this that humanity has been suffering for the want of petticoated physicians; that masculine talent is not suited to medical necessities. Female medical education is a "necessity of the age;" that public sentiment, which a century or two ago drove the female midwife from the practice of obstetrics, and substituted the female accoucheur, was not an advance, but a retrograde movement; and a "refined civilization" requires that we should go back to the practice of our ancestors. Refined civilization! that must indeed be refinement which would subject the tender nature of women to the hardening influences of a medical life, and crush out of her all those gentle impulses which make her what she is—make a business and trade of her sympathies, and place the knife of the surgeon in the soft hand which was wont only to soothe and comfort.

"They find the demand for female physicians wide-spread and increasing, and regard the study and practice of medicine as peculiarly adapted to the nice perceptions of women, and the tenderness and refined graces of her nature."

Do they? Then they find what in our blindness we have never discovered. To judge from the only female physician whose success we have had the opportunity of judging, we should opine that the demand was not so large as to be overwhelming. She came to this city three years ago, lived through by some mysterious process to us unknown, had no patients that we ever heard of, shut up her office and resorted to woman's natural occupation—looking up a husband—and, having succeeded in that, left the city.

But the "nice perceptions" of women so peculiarly adapt her to practice. The phrase is cunningly used. Women, perhaps, excel in perceptive faculties, but we have never known that they prospered especially in logic. A woman rarely stops to reason. It is a beautiful element in her character that she is impulsive, and learns all she ever knows of a subject at the first glance. This intuitive peception is a child-like element of character which produces sometimes really wonderful results, but something more than all that is necessary to the physician. Keen perceptions are too often linked with a timid nature, and the female physician, while she would often appreciate fully the danger of her patient, would lack in that fortitude, calm judgment, and indifference to the opinions of others, which are essential to success in practice. If there is any occupation which requires certain elements of character not usually coupled with the adjective *feminine*, such as prompt decision, physical endurance, disregard of personal comfort, courage, and coolness under excitement, it is the profession of medicine.

"The tenderness and refined graces of her nature" (woman's) will not be especially illustrated in assuming the duties of the accoucheur.

The forceps, the murderous perforator, and that blunt hook are neither tender, refined, nor graceful; and though we might respect the woman who could use them well and manfully, it would be the same very distant respect which we cherish for an accomplished female tight-rope dancer. It is absurd to suppose a life passed among old sores, acrid discharges and unhealthy expectoration, to be suited to the gentle nature of woman. The hold which woman has upon male humanity is not founded in the possession on her part of the strong, heroic element; and so soon as we learn to look upon them as other than gentle and kindly, needing our protection, so soon do we destroy every element of that chivalry which has ever been woman's surest defence, and has done so much to humanize and soften the masculine character.

Change these relations, place the two sexes in competition, and the feebler is no longer the idol to which every worthy offering is brought, but simply a fellow-struggler in the world-wide game of "devil-take-the-hindmost." The woman who places herself in such a position is a fool.

"They consider that woman, as a wife and mother, pre-eminently *needs* a clear understanding of the functions of the human body and the means of preserving health; and that high-toned and intelligent female physicians, from their relations to their sex, must be most important instrumentalities in imparting such knowledge, where it is most needed and will do the most good."

This is true enough. Whether as wife and mother, or as school girl, woman does need to know that out-door air and exercise are a part of her proper "functions," and that cotton, whalebone, and curled-hair bosoms are poor substitutes for that natural rotundity of figure and softness of outline which form her chiefest charm, as evidence of her adaptation to the one greatest purpose of her being. Whether we must educate a bevy of female apostles of natural hygiene for this express purpose, is another question. We find no difficulty, either in our own constitutional bashfulness, or in the modesty of our feminine hearers, in preaching the gospel of nature with due earnestness and unction. Our success to be sure is indifferent, for fashion is a stronger argument than we can bring, but we fear the female preacher will suffer from the same difficulty.

"It is well known that there is a vast amount of suffering among women, which is left without relief from the shrinking delicacy of its victims, and it is therefore a demand of humanity that women should be put in possession of the requisite knowledge to administer the required treatment in such cases."

There is more suffering left unrelieved from ignorance on the part of the practitioner than any other cause. This "shrinking delicacy" is a myth. No sensible woman allows it to interfere with her health; and those who are not sensible can usually be brought to see the necessity for setting aside an artificial modesty, and doing what is fit and necessary to be done.

"They also desire a scientific medical education for woman, because it will furnish her honorable and profitable *employment*—giving her a new sphere of usefulness and happiness, where duty and the sympathies of her nature lead her: in the chambers of the sick and the suffering."

The motive is a good one; the means of carrying it out anything but good. In the progress of our civilization it has become evident that woman needs and is justly entitled to a lager compensation for the services she renders. We employ a needle-woman in an occupation requiring education, taste, judgment, and true perceptions of beauty in form and color, and pay her less for the day's labor than we give a ragged street urchin for running an errand. It is wrong, but if one wish to degrade woman, let him exhibit her incompetence by placing her in the practice of a profession to which she is unsuited, as well by her best qualities as her poorest—her strength in her weakness.

Here we leave the subject. We have hardly touched the real argument, contenting ourselves with a concise reply to the appeal of the corporators. We sincerely trust that they will fail in procuring the $50,000 which they ask to place themselves on a substantial basis. It would almost inevitably be one of the most mischievous expenditures conceivable. An endowment to enable women—to do what? To sit in dissecting rooms and cut up dead humanity, and to forget all that which makes them feminine. To watch, unmoved by any useless pity, the writhings of the victim of the surgical operation; to go out to the world prepared to do this thing themselves; to leave the gay world of dance, and song, and flowers, and dress, to which their nature adapts them, and in which they are beautiful, and devote themselves to the study of ulcerations and eruptions; to look with critical eye on scald-head and milk crust; to bleed, and blister, and purge, puncture abscesses, and split up carbuncles. Faugh! protect us from such women!—*Buffalo Med. Journ.*—*Am. Med. Gazette.*

